# Effects of coaching supervision, mentoring supervision and abusive supervision on talent development among trainee doctors in public hospitals: moderating role of clinical learning environment

**DOI:** 10.1186/s12909-015-0407-1

**Published:** 2015-08-13

**Authors:** Anusuiya Subramaniam, Abu Daud Silong, Jegak Uli, Ismi Arif Ismail

**Affiliations:** 1 Department of Professional Development & Continuing Education, Faculty of Educational Studies, Universiti Putra Malaysia, 43400 Serdang, Selangor Malaysia; 2 Faculty of Business & Management, Asia Pacific University of Technology & Innovation (APU), Technology Park Malaysia, Bukit Jalil, 57000 Kuala Lumpur, Malaysia; 3 Department of Defence Human Resource Management, Faculty of Defence Studies & Management, Universiti Pertahanan Malaysia, Kem Sungai Besi, 57000 Kuala Lumpur, Malaysia

**Keywords:** Coaching supervision, Mentoring supervision, Abusive supervision, Clinical learning environment, Talent development, Professional and medical competencies, Medical education, Trainee doctors, Public hospitals, Malaysia

## Abstract

**Background:**

Effective talent development requires robust supervision. However, the effects of supervisory styles (coaching, mentoring and abusive supervision) on talent development and the moderating effects of clinical learning environment in the relationship between supervisory styles and talent development among public hospital trainee doctors have not been thoroughly researched. In this study, we aim to achieve the following, (1) identify the extent to which supervisory styles (coaching, mentoring and abusive supervision) can facilitate talent development among trainee doctors in public hospital and (2) examine whether coaching, mentoring and abusive supervision are moderated by clinical learning environment in predicting talent development among trainee doctors in public hospital.

**Methods:**

A questionnaire-based critical survey was conducted among trainee doctors undergoing housemanship at six public hospitals in the Klang Valley, Malaysia. Prior permission was obtained from the Ministry of Health Malaysia to conduct the research in the identified public hospitals. The survey yielded 355 responses. The results were analysed using SPSS 20.0 and SEM with AMOS 20.0.

**Results:**

The findings of this research indicate that coaching and mentoring supervision are positively associated with talent development, and that there is no significant relationship between abusive supervision and talent development. The findings also support the moderating role of clinical learning environment on the relationships between coaching supervision-talent development, mentoring supervision-talent development and abusive supervision-talent development among public hospital trainee doctors. Overall, the proposed model indicates a 26 % variance in talent development.

**Conclusion:**

This study provides an improved understanding on the role of the supervisory styles (coaching and mentoring supervision) on facilitating talent development among public hospital trainee doctors. Furthermore, this study extends the literature to better understand the effects of supervisory styles on trainee doctors’ talent development are contigent on the trainee doctors’ clinical learning environment. In summary, supervisors are stakeholders with the responsibility of facilitating learning conditions that hold sufficient structure and support to optimise the trainee doctors learning.

## Background

The success of healthcare systems worldwide hinges on the development and competence of its doctors [1]. According to evidence-based management theory, doctors possess craft that can be learned/developed with appropriate guidance through practice and experience [2]. Housemanship provides trainee doctors the chance to perform required medical procedures and undertake clinical practice under supervision [1]. Existing studies have emphasised medical education and training, illustrating a cognitive ‘schooled’ approach that emphasises on competence-based development for young doctors [2]. The indicators of competence in medicine are derived to evaluate the qualities necessary for a medical practitioner to function effectively, which includes professional and medical competencies [3]. Nevertheless, controversy exists on how to develop these competencies [1]. These set of competencies are widely known as talent. In the existing state of the art the trainee doctors’ talent development has not been identified and investigated extensively.

In this study, talent development refers to the competency development for medical practitioners that is geared towards producing competent professionals with necessary skills for medical practice. Effective talent development approach requires robust supervision [4]. In the healthcare environment, supervisors are portrayed as role models for young doctors. Thus, healthcare supervisors should portray qualities that include the abilities to communicate, inspire, to demonstrate integrity, honesty and consistency [5]. Prior scholars asserted that supervisory styles have significant implications for trainee doctors’ talent development [6]. For instance, coaching [7], mentoring [7] and abusive supervision [8] have been utilised to establish the relationship between supervision and performance. The causal relationships between these aspects lead towards talent development in healthcare setting. Through supervisory coaching, supervisors who pass on accumulated “wisdom” to their mentees led to the development of young talents [9]. Mentoring supervision has been described as “an experienced person who goes out of his/her way to help a mentee set important life goals and develop the skills to reach them” [10]. On the other hand, abusive supervision leads to individuals being exhausted and incompetent [8, 11]. Despite the contrasts in the different supervision styles, few studies have provided support linking supervisory styles with talent development among public hospital trainee doctors.

For trainee doctors, their competency level lies in high-quality supervised training that provides exposure to various medical cases, treatment scenarios and diagnostic tools—all of which indicate a favourable clinical learning environment [12]. A favourable clinical learning environment is termed as one that provides organisational and socio-cultural interactions that support trainee doctors’ entry to the formal and technical elements of the environment [13]. Such an environment provides organised activities, resources and chances for practice [13]. Undeniably, a favourable clinical learning environment is one of the most essential aspects of the quality of medical training [14]. Prior work in this area asserted that a favourable clinical learning environment enhances the effects of coaching [15] and mentoring supervision [16] on trainee doctors’ talent development. Similarly, the relationship between abusive supervision and talent development is contingent on a favourable clinical learning environment [17]. Despite their importance, researches on the moderating effects of clinical learning environment in the following relationships among public hospital trainee doctors are limited: between coaching supervision-talent development, mentoring supervision-talent development and abusive supervision-talent development.

Prior scholarly efforts in talent development in healthcare environment tend to focus on conceptual ideas that often lack empirical evidence. It is clear that development of trainee doctors’ competencies (talent development) is dependent on high-quality supervised training [16]. This can be further enhanced by generating a favourable clinical learning environment [18]. Nevertheless, there is lack of studies that integrate the concepts of supervisory styles with talent development and examine whether a favourable clinical learning environment influences these relationships. In this regard, the contribution of this study is significant.

### Hypotheses development

The self-determination theory provides useful perspectives for understanding coaching [19]. At the macro level, the theory determines the kind of environment that is needed if individuals are to "do well" and "feel good" throughout their lives [20]. On a micro level, the theory can assist practitioners to understand the importance of workplace interactions which are inclusive of the process relating to interpersonal conditions for achieving optimal growth and generating development [19]. The use of skills includes active listening, emotional intelligence, empathy other signs that augments the developmental processes towards mentees’ growth [21]. Thus, the developmental processes demonstrated in self-determination theory will assist towards an understanding of how supervisees can enhance their confidence and generate better interpersonal skills, as well as enable them to cope with changes and difficulties in their path [22]. Pertaining to the medical context, coaching supervision aids trainee doctors towards developing their competencies [23]. This suggests the following:

*H1: Coaching supervision is positively related to talent development.*

Mentoring supervision facilitates the mentee transition from the learner status towards being an expert [24]. Based on social learning theory [25], followers tend to imitate the behaviour they see in their supervisors whom they respect and admire. Therefore, observations of the effective supervision, consultation, empowerment and confidence in own capabilities are essential to be successful [24]. These observations and attributes indicate that mentoring supervision can facilitate skill learning professional development through the use of these concepts. Career development theory describes mentoring supervision as an essential phase to enhance supervisees’ personal and professional life by generating quality relationships and career success [26]. Thus, mentoring supervision is an important development tool for supervisees and at the same time creates a fulfilling experience to those serving as mentors [7]. Mentoring supervision, an established means of professional development, is widely used in medicine [27]. For instance, reflection is an important part of the mentoring process which can provide a fresh impetus to the personal and professional development of doctors [27]. This suggests the following:

*H2: Mentoring supervision is positively related to talent development.*

With regards to social learning, supervisees imitate the negative behaviour as observed from the supervisor in the form of workplace bullying [28]. This describes the association between negative supervision and supervisees outcomes. Social learning and trickled down models can have high influence when there is high power distance between the leader and followers [28]. This not only increases the emergence of negative supervision but also worsens the consequences of negative supervision as the mentee may feel he/she cannot react to the behaviour [29] and thus which in turn may lead to feeling of helplessness [30]. Thus, it is recommended that abusive supervisors be removed since abusive behaviours do not nurture future talent [31]. This suggests the following:

*H3: Abusive supervision is negatively related to talent development.*

Supervising adult learners assumes the role of a novice practitioner which implies that supervising in its broadest sense must give way to facilitating learning in a supportive environment [32]. Coaching supervision is a trainee driven process assisted by encouragement from coach whose knowledge and skills towards encouraging understanding and utilisation of the newly obtained knowledge and skills [15]. This indicates that coaching supervision facilitates competency development and raises confidence in trainee doctors’ abilities. Nevertheless, the environment in which trainee doctor is associated can influence their talent development as learning in workplace atmosphere is more effective and likely to provoke knowledge productivity [33]. This reveals the potential role of clinical learning environment as a moderator in the relationship between coaching supervision and talent development. This suggests the following:

*H4: Clinical learning environment moderates the relationship between coaching supervision and talent development.*

Social learning theory supports mentorship and healthcare learning that recognises the significance of participation to support learning [34]. Mentoring is a protected interaction in which learning takes place through analysis, assessment, reassessment and practice exposure, conditions, coupled with difficulties, errors and accomplishments [35]. These characteristics facilitate the supervisees’ talent development. The quality of the interaction is important towards a successful outcome [35]. However, to facilitate mentoring supervision and encourage successful outcomes, certain environmental condition must prevail, which is the provision of a well prepared, flexible, involved and generally, a favourable clinical learning environment [35]. A favourable clinical learning environment prevails as mentors generate a learning environment by guiding their mentees and provide them with resources and suggestions [35]. Guidance is provided if they encounter any problems. This in turns assists mentees to encounter any barriers and increases their capacity to anticipate such problems [35]. Thus, it is argued that this favourable learning environment influences the strength of the relationship between mentoring supervision and talent development. This suggests the following:

*H5: Clinical learning environment moderates the relationship between mentoring supervision and talent development.*

With regards to social exchange theory, trainees with feeling of hurt are more likely to result in negative consequences such as serious medical error [11, 36]. Nevertheless, it is noted that a favourable clinical learning environment alters such adverse consequences [37, 38]. Carl Rogers, who pioneered theories on counselling, believes in a favourable clinical learning environment as it makes trainee doctors feel that they are able to express themselves in an open manner without the fear of reproachment [39]. In contrast, unfavourable clinical environment with inadequate infrastructure/personnel can impede trainee doctors’ talent development [17]. The negative experiences due to mistreatment are lessened when trainee doctors encounter favourable clinical learning environment, which enhances their talent development. Therefore, it is argued that a favourable clinical learning environment influences the effects of abusive supervision on trainee doctors’ talent development. This suggests the following:

*H6: Clinical learning environment moderates the relationship between abusive supervision and talent development.*

## Methods

The study was carried out in Malaysia—an emergent Southeast Asian country. Participants were trainee doctors from six Malaysian public hospitals in the Klang Valley area. Prior approval was attained from the Ethics and Research Committee of Ministry of Health Malaysia to conduct research, which eased access to six hospitals. A questionnaire-based critical survey was conducted among the individual trainee doctors. The Human Resource (HR) Training Unit at each hospital assisted in the distribution of the questionnaires. The trainee doctors were given the option to refuse participation. It was made clear that returning the questionnaire after completion was considered as informed consent for participation in the study. Out of 450 distributed questionnaires, 355 were completed and had usable responses.

### Measures

Coaching supervision was measured using the 11-item scale [40]. A 5-point rating scale (1 = *Do not facilitate*, 5 = *Highly facilitate*) was utilised to measure the extent to which the trainee doctors perceived that their immediate supervisors had the following behaviour(s) in relation to coaching supervision that in turn could assist in facilitating their talent development. Mentoring supervision was measured using a 15-item scale [41], composed of three dimensions: (1) *Psychosocial support*, (2) *Career development* and (3) *Role modelling*. The extent to which the trainee doctors perceived that their immediate supervisor in relation to mentoring supervision and behaviour was measured on a 5-point Likert scale (1 = *Do not facilitate,* 5 = *Highly facilitate*). Abusive supervision scale was measured using a 15-item scale [42]. A 5-point rating scale (1 = *Do not facilitate,* 5 = *Highly facilitate*) was utilised to measure the extent to which the trainee doctors perceived that their immediate supervisor possess attributes indicating abusive behaviour or supervision that could hinder their talent development*.* Conversely, the rating scale is also used to identify attributes that assist in facilitating positive development in the trainee doctor experience in the medical housemanship.

In this paper, clinical learning environment was measured using the 10-item scale [13] comprised of three dimensions: (1) *Conditions for Learning*, (2) *General Learning Activities and Resources* and (3) *Opportunities to perform rotation-specific clinical skills and assessment*. The extent to which the trainee doctors agree with the statements pertaining to the aspect of clinical learning environment that could facilitate talent development was measured on 5-point Likert scale (1 = *Do not facilitate,* 5 = *Highly facilitate*). The measurement scale for talent development comprised 13 items [3], inclusive of a number of competence items. Each component of professional and medical competencies computes several aspects of competencies needed by trainee doctors for independent practice. These are classified into three dimensions: (1) *Clinical competence,* (2) *Communication competence* and (3) *Personal and professional competence*. Each of the items relating to competencies was rated on a 5-point rating scale (1 = *Do not facilitate,* 5 = *Highly facilitate*).

### Data analysis

In order to assess the extent of common method variance, Harman’s one factor test [43] was carried out. All the scale items were inserted into an unrotated factor analysis to identify whether a single factor accounted for the majority of covariance among the constructs. This process results in a seven factor (Eigenvalues greater than 1.0) solution, whereby the first factor accounted for 33.72 % of the variance, which is less than 50 %. This indicates that the common method bias does not appear to be a serious problem in the study.

### Statistical analysis

SPSS 20.0 was employed to analyse preliminary data, while the rest used Amos 20.0 for SEM as it quantifies the theoretical relationships of constructs that combines regression and factor analysis [44]. The proposed model was tested by utilising a two-stage approach (measurement and structural model assessment).

## Results

### Measurement model assessment

The measurement model is analysed using Confirmatory Factor Analysis (CFA) and evaluated in two conditions: (1) unidimensionality for each scale and (2) reliability and validity of each constructs.

In order to achieve unidimensionality, a factor loading of 0.50 and above on a specified factor is considered acceptable [45] and is used as the threshold value in this research. Also, items with weak factor loading on the hypothesised factors were removed from the scale, resulting in a unidimensional scale (as per Table 1).

**Table 1:** Measurement model evaluation

Constructs	Items	Standardized loading (λ)	Cronbach’s alpha	CR
CS	C1 to C11	(Between 0.70 and 0.81)	0.94	0.95
MS	PSY1 to PSY5	(Between 0.80 and 0.89)	0.93	0.97
	CD7 to CD11	(Between 0.79 and 0.89)		
	RM13 to RM15	(Between 0.78 and 0.97)		
AS	AS1 to AS15	(Between 0.74 to 0.95)	0.98	0.98
CLE	CL1 to CL6	(Between 0.78 and 0.89)	0.96	0.96
	GLAR7, GLAR10	(Between 0.79 and 0.83)		
	OPP8, OPP9	(Between 0.88 and 0.92)		
TDEV	CLI1, CL2, CL4	(Between 0.72 and 0.86)	0.92	0.94
	COMC6, COMC7	(Between 0.79 and 0.86)		
	PER8, PER11, PER12, PER13	(Between 0.73 and 0.80)		

*CS* coaching supervision, *MS* mentoring supervision, *AS* abusive supervision, *CLE* clinical learning environment, *CL* conditions for learning, *GLAR* general learning activities and resources, *OPP* opportunities to perform rotation-specific clinical skills and assessment, *TDEV* talent development, *CL* clinical competence, *COMC* communication competence, *PER* personal and professional competence; *CR* composite reliability

The constructs of the study were assessed by utilising the following criteria to achieve reliability and validity:

- Cronbach’s [46] alpha indicator must be greater than 0.70 for each construct to support reliability;
- Composite reliability should be equal to or greater than 0.60 [47];
- Construct validity obtained from goodness-of-fit indices [45];
- Average extracted variance (AVE) should be equal to or greater than 0.50 to support convergent validity [47]; and
- AVE must be greater than the squared correlation estimates of among the constructs to support discriminant validity [47].

Tables 1 and 2 indicate that the measures used in this research are within the acceptable levels, thus supporting the reliability and validity of the constructs used. The output of CFA for the measurement model is as follows: chi-squared statistic divided by the degrees of freedom (CMIN/df), 1.813; the comparative fit index (CFI), 0.934; and the root mean square error of approximation (RMSEA), 0.048, which showed a good fit [(CMIN⁄ df < 3.0); CFI > 0.90; RMSEA < 0.06] [45].

**Table 2:** Descriptive statistics, reliability and validity results

Constructs	Mean	Std Dev	95 % CI	CS	MS	AS	CLE	TDEV	AVE
CS	3.91	0.723	(3.83-3.98)	1.00	0.608^a^				0.62
MS	3.61	0.763	(3.53-3.69)	0.782**	1.00	0.014			0.73
AS	2.25	1.146	(2.13-2.37)	−0.339**	−0.120*	1.00	0.077		0.76
CLE	3.85	0.731	(3.77-3.92)	0.520**	0.489**	−0.277**	1.00	0.184	0.71
TDEV	3.85	0.582	(3.79-3.92)	0.425**	0.396**	−0.183**	0.429**	1.00	0.63

*Std Dev* standard deviation; *95 % CI* 95 % confidence interval, *CS* coaching supervision, *MS* mentoring supervision, *AS* abusive supervision, *CLE* clinical learning environment, *TDEV* talent development, *AVE* average variance extracted

*p < 0.05;**p < 0.01

^a^Values above the diagonal are the squared correlations

AVE of each construct and the squared correlation estimates are illustrated in Table 2, together with mean, standard deviation and 95 % confidence interval of each construct. From the values of AVE and squared correlation estimates, it is evident that the criteria for convergent and discriminant validities of each construct is satisfied. Moreover, with respect to the mean value, a score of 3.5 or more indicates high agreement with a particular criterion, whereas a score of more than 2.5 but less than 3.5 indicates moderate agreement. Similarly, a score less than 2.5 indicate low agreement with a criterion. Based on the mean values of the constructs, public hospital trainee doctors have high preference for coaching supervision (mean = 3.91) and mentoring supervision (mean = 3.61), but low preference for abusive supervision (mean = 2.25), as well as an optimal clinical learning environment (mean = 3.85) and talent development (mean = 3.85).

### Structural model assessment

A structural equation model was run in AMOS to examine the path diagram. Fit statistics for the structural model was: CMIN/df, 2.078; CFI, 0.912; and RMSEA, 0.055, showed a good fit. Further results of the fully tested structural model are shown in Table 3.

**Table 3:** Results of the structural model

Path From → To	UPC/SPC/p-value
CS → TDEV	0.167/0.200/0.001***
MS → TDEV	0.129/0.121/0.019*
AS → TDEV	−0.013/-0.027/0.597
CLE → TDEV	0.262/0.316/0.001***
CS → CLE	0.391/0.388/0.001***
CS*CLE → TDEV	0.119/0.296/0.002**
MS → CLE	0.212/0.166/0.001***
MS*CLE → TDEV	−0.106/-0.259/0.006**
AS → CLE	−0.096/-0.170/0.001***
AS*CLE → TDEV	0.084/0.170/0.001***

*CS* coaching supervision, *MS* mentoring supervision, *AS* abusive supervision, *CLE*, clinical learning environment, *TDEV* talent development, *UPC*, un-standardised path coefficient, *SPC*, standardised path coefficient

*p < 0.05; **p < 0.01; ***p < 0.001

Table 3 reveals that the path between coaching supervision and talent development (β = 0.200, *p* = 0.001), as well as mentoring supervision and talent development (β = 0.121, *p* = 0.019). These values are statistically significant, thus hypotheses *H1* and *H2* is supported. Nevertheless, the results do not establish support for hypothesis *H3,* where the path between abusive supervision and talent development is not statistically significant (β = −0.027, *p* = 0.597).

To test the moderating effects of clinical learning environment several constructs including the exogenous constructs (coaching, mentoring and abusive supervision), the moderating construct (clinical learning environment) and the interaction term [coaching supervision x clinical learning environment; mentoring supervision x clinical learning environment; and abusive supervision x clinical learning environment] were regressed on endogenous construct (talent development). Table 3 shows the output of the analysis process. Since the interaction term implies a statistically significant amount of variance in the endogenous construct, a moderator effect is present. This indicates that clinical learning environment moderates the relationship between (1) coaching supervision and talent development (β = 0.296, *p* = 0.002), (2) mentoring supervision and talent development (β = −0.259, *p* = 0.006) and (3) abusive supervision and talent development (β = 0.170, *p* = 0.001). With these values, hypotheses *H4*, *H5* and *H6* are supported. Overall, the model explains 26 % of the variance in talent development.

**Fig. 1:**
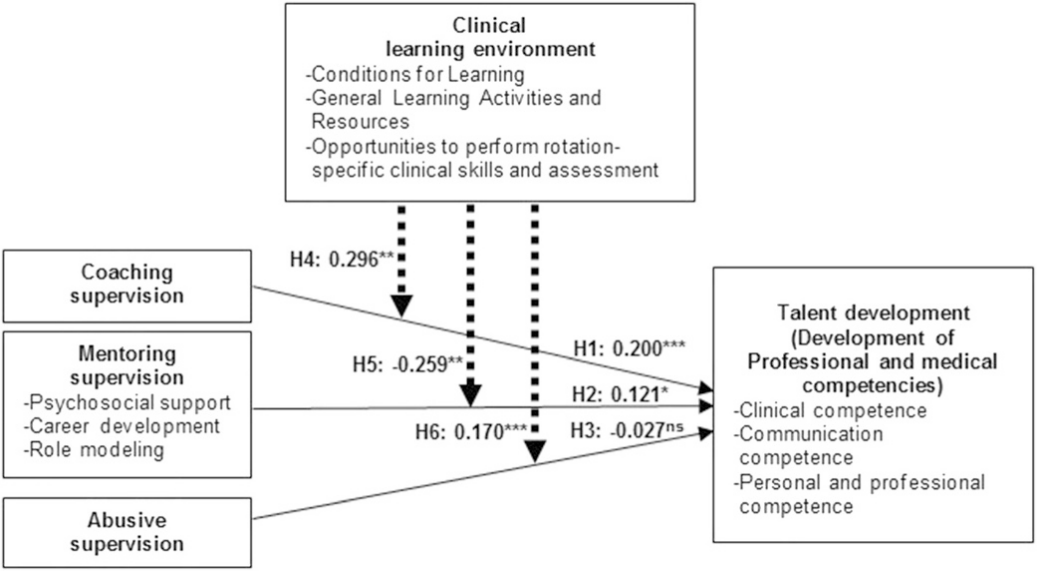
Structural Model Results. Note. H = Indicates all the hypotheses cited in this study; ns = not significant; *p < 0.05; **p < 0.01;***p < 0.001

A summary of the findings is given in Fig. 1.

## Discussion

The findings of the study are consistent with prior research [9, 48] that indicate coaching supervision facilitates talent development. Thus, healthcare professionals should conduct training and educational programmes for supervisors on how to serve as coaches. As revealed by prior work [16], the findings also show that the relationship between coaching supervision and talent development is stronger when trainee doctors perceive a favourable clinical learning environment. This observation in the prior work gives credence to the finding and relevance of this effort. Thus, healthcare administrators should provide a favourable clinical learning environment to enhance trainee doctors’ talent development. This can be conducted by providing trainee doctors the chance to repeat the learned skills and in different situations and contexts (mixed practices) until they become competent. Furthermore, continuous feedback should be provided as this will help to improve their competency level by understanding the outcomes of their performance.

As noted in prior research [10, 49], mentoring supervision facilitates talent development. Thus, mentoring supervision can be incorporated into housemanship by training supervisors on how to develop their interpersonal capabilities, relationship skills and conflict resolution assistance. Furthermore, trainee doctors can be encouraged to reflect on their learning by allowing them to conduct case presentations and constantly provide feedback. Results further indicate that the interaction between mentoring supervision and talent development is stronger when trainee doctors perceive a favourable clinical learning environment. Thus, supervisors can provide trainee doctors with relevant chances to observe patients with variations of clinical problems.

Results of this study indicate that coaching supervision is a contributing factor towards talent development compared to mentoring supervision. Supervisors could coach trainee doctors by selecting tasks that are suitable to their capabilities, offer critical evaluation of this capability, dispensing advice, formulating a process to execute tasks and developing a structured way to address weaknesses.

The findings of this study are consistent with that of prior research work [50] that show abusive supervision does not have any direct effect on talent development. Supervisees were found to be highly responsive towards negative aspects of external context, which tends to have strong effect on attitudes and behaviour compared to positive contextual aspects [50]. Trainee doctors’ supervisors are likely to retaliate or emulate their supervisors with mistreatment to enhance supervisees’ talent development. Nevertheless, negative experiences due to supervisory abuse are lessened when trainee doctors encounter a favourable clinical learning environment, which could enhance their talent development. For this reason, a favourable clinical learning environment for trainee doctors is necessary towards nurturing and motivating them to apply their potential in enhancing their talent development [51]. Furthermore, public hospital administrators should conduct training programmes for trainee doctors to eliminate problems that arise during the housemanship tenure and encourage them to become confident in facing negative consequences in their learning environment. Additionally, a grievance or ombudsman system [52] for trainee doctors is needed to prevent supervisory abusive during housemanship.

These findings imply that clinical learning environment plays an important role on supervisory styles in facilitating talent development. Thus, public hospital administrators should support trainee doctors’ talent development by providing sufficient specialty services, mixture of cases and specialists to ensure effective supervision. It is essential to monitor trainee doctor progress through training programmes as well as feedback and appropriate opportunities to maximise learning [53].

## Conclusion

From the theoretical standpoint and theory building, this study has contributed toward the work on talent development among medical practitioners. This study has attempted to improve the understanding on the supervisory styles that can facilitate talent development among public hospital trainee doctors. It is observed that the interactional effects of clinical learning environment are essential for the delivery of quality supervisory training thus enabling public hospital trainee doctors’ talent development. This study and findings also deepens our understanding of the underlying mechanisms that are responsible for the direction/strength of the relationship between supervisory styles and talent development.

In summary, supervisors have the responsibility of providing learning conditions that hold sufficient structure and support trainee doctors’ learning. The assessment by the supervisor of trainee doctor development level shall help in identifying the optimal learning environment for trainee doctors. Furthermore, public hospital administrators can develop training modules to address trainee doctors’ needs and generate an environment that will encourage them to apply their skills.

Given the limitations of the cross-sectional design, future studies should consider longitudinal data to establish causal relationships and to identify developmental changes over time among these constructs. The sample of this study included public hospital trainee doctors only, it follows that due the similar characteristics and scenario including private hospital trainee doctors is likely to present interesting relationships and differences. Finally, the model explains 26 % variance in talent development. The percentage of the explained variance of trainee doctors’ talent development could in fact be related to constructs other than the studied ones. Future research could design model that incorporate the differences in the constructs.

## References

[1] Spilg E, Siebert S, Martin G (2012). A social learning perspective on the development of doctors in the UK National Health Service. Soc Sci Med.

[2] Pfeffer J, Sutton RI (2006). Evidence-based Management. Harv Bus Rev.

[3] McGill DA, Van Der Vleuten CPM, Clarke MJ (2013). A critical evaluation of the validity and the reliability of global competency constructs for supervisor assessment of junior medical trainees. Adv Health Sci Educ.

[4] Sweem SL (2009). Leveraging Employee Engagement through a Talent Management Strategy: Optimizing Human Capital through Human Resources and Organization Development Strategy in a Field Study [Dissertation].

[5] Pope R. Staff Behaving Badly. 2009. http://www.cmf.org.uk/publications/content.asp?context=article&id=2193. Accessed 7 June 2012.

[6] Akerjordet K, Severinsson E (2007). Emotional Intelligence: A review of the literature with specific focus on empirical and epistemological perspectives. J Clin Nurs.

[7] Kavanaugh J, Duffy JA, Lilly J (2006). The relationship between job satisfaction and demographic variables for healthcare professionals. Manag Res News.

[8] Ayree S, Chen ZX, Sun L, Debrah YA (2007). Antecedents and outcomes of abusive supervision: test of a trickle-down model. J Appl Psychol.

[9] Onyemah V (2009). The effects of coaching on salespeople's attitudes and behaviors: A contingency approach. Eur J Market.

[10] Russell JEA, Adams DM (1997). The changing nature of mentoring in organizations: An introduction to the special issue on mentoring in organizations. J Vocat Behav.

[11] Yun-xia F, Liu J (2010). Abusive Supervision: An Interactive Model of Human Resource Management.

[12] Teunissen P, Scheele F, Scherpbier A, Van der Vleuten CPM, Boor K, Van Luijk SJ, Van Diemen- Steenvoorde C (2007). How residences learn: qualitative evidence for the pivotal role of clinical activites. Med Educ.

[13] Emilia O, Bloomfield L, Rotem A (2006). Replication of a clinical learning environment survey for junior medical officers: a study of medical students in an Indonesian hospital. Focus Health Professional Education.

[14] Maudsley RF (2001). Role models and the learning environment: essential elements in effective medical education. Acad Med.

[15] Fuimano J (2007). Coaching and mentoring for the new work force. Gastroenterol Nurs.

[16] Sheehan DC (2011). Learning and Supervision in Internship: A sociocultural framework for understanding learning and supervision in medical internship [Dissertation].

[17] Gourevitch MN, Malaspina D, Weitzman M, Goldfrank LR (2008). The public hospital in American medical education. J Urban Health.

[18] Daelmans H, Hoogenboom R, Donker A, Scherpbier A, Stehouwer C, Van Der Vleuten C. Effectiveness of clinical rotations as a learning environment for achieving competences. Med Teach. 2004;26:305–312. 10.1080/01421590410001683195 15203842

[19] Spence GB, Oades LG (2011). Coaching with self-determination in mind: Using theory to advance evidence-based coaching practice. Int J Evid Based Coach Mentor.

[20] Ryan RC, Deci EL (2000). Self-determination theory and the facilitation of intrinsic motivation, social development, and well-being. Am Psychol.

[21] Keyes CLM, Haidt J (2003). Flourishing: Positive psychology and the life well-lived.

[22] Stradling HA (2008). Using coaching and mentoring skills to become an effective educational supervisor. Found Years.

[23] Frederiksen CH, Donin J, Koschmann TD, Kelson AM. Investigating Diagnostic Problem Solving in Medicine through Cognitive Analysis of Clinical Discourse. In Proceedings of the 2004 Annual Meeting of the Society for Text & Discourse: 1–4 Aug 2004; Chicago, IL. http://www.mcgill.ca/files/edu-acsrg/CogAnalysisofClinicalDiscourse.pdf. Accessed 30 January 2013.

[24] Pop RS (2011). Mentoring nurse practitioners in a hospital setting [Dissertation].

[25] Bandura A (1977). Social Learning Theory.

[26] Gedde C, Strickland B (1984). From plateaus to progress: a model for career development. Training.

[27] Taherian K, Shekarchian M (2008). Mentoring for doctors. Do its benefits outweigh its disadvantages?. Med Teach.

[28] Schyns B, Schilling J (2013). How bad are the effects of bad leaders? A meta-analysis of destructive leadership and its outcomes. Leader Q.

[29] Tepper BJ (2007). Abusive supervision in work organizations: Review, synthesis, and research agenda. J Manag.

[30] Seligman MEP (1975). Helplessness: On depression, development and death.

[31] Boddy CR (2011). Corporate psychopaths, bullying and unfair supervision in the workplace. J Bus Ethics.

[32] Clinical Teaching and Clinical Instruction Guidelines. 2014. http://www.med.monash.edu.au/radiography/docs/section3-clinical-teaching-and-instruction.pdf. Accessed 24 February 2014.

[33] Schroeter K (2008). Competence Literature Review.

[34] Billett S (2002). Towards a workplace pedagogy; guidance, participation and engagement. Adult Educ Q.

[35] McKimm J, Jollie C, Hatter M (2007). Mentoring: Theory and practice.

[36] Paice E, Rutter H, Wetherell M, Winder B, McManus IC (2002). Stressful incidents, stress and coping strategies in the pre-registration house officer year. Med Educ.

[37] Huang MH. What Makes Abusive Supervision Survive? Self-Efficacy and the Perception of Authenticity Alleviate the Negative Consequences of Abusive Supervision. 2012. http://thesis.topco-global.com/TopcoTRC/2013_Thesis/H0088.pdf. Accessed 24 February 2014.

[38] Paltridge D (2006). Prevocational medical training in Australia: where does it need to go?. Med J Aust.

[39] Walsh D (2010). The Nurse Mentor’s Handbook: Supporting Students in Clinical Practice.

[40] Arnold JA, Arad S, Rhoades JA, Drasgow F (2000). The empowering leadership questionnaire: the construction and validation of a new scale for measuring leader behaviours. J Organ Behav.

[41] Scandura TA, Ragins BR (1993). The effects of sex and gender role orientation on mentorship in maledominated occupations. J Vocat Behav.

[42] Tepper BJ (2000). Consequences of abusive supervision. Acad Manage J.

[43] Podsakoff P, Organ D (1986). Self-reports in organizational research: problems and prospects. J Manag.

[44] Tabachnick BG, Fidell LS (1996). Using multivariate statistics.

[45] Hair J, Black W, Babin B, Anderson R, Tatham R (2006). Multivariate data analysis.

[46] Cronnbach LJ (1951). Coefficient Alpha and the Internal Structural of Tests. Psychometrica.

[47] Fornell C, Larcker DF (1981). Evaluating structural equation models with unobservable variables and measurement error. J Market Res.

[48] Hamlin RG, Ellinger AD, Beattie RS (2009). Toward a Profession of Coaching? A Definitional Examination of ‘Coaching’, ‘Organization Development’, and ‘Human Resource Development. Int J Evid Based Coach Mentor.

[49] Hezlett SA, Gibson SK (2005). Mentoring and Human Resource Development: Where We Are and Where We Need to Go. Adv Dev Hum Res.

[50] Baumeister RF, Bratslavsky E, Finkenauer C, Vohs KD (2001). Bad is stronger than good. Rev Gen Psychol.

[51] Degen C, Weigl M, Glaser J, Li J, Angerer P (2014). The impact of training and working conditions on junior doctors’ intention to leave clinical practice. BMC Med Educ.

[52] Boswell WR, Olson-Buchanan JB (2004). Experiencing mistreatment at work: The role of grievance filing, nature of mistreatment and employee withdrawal. Acad Manage J.

[53] Premadasa IG, Shehab D, Al-Jarallah KF, Thabib L (2007). Confidence in performing core clinical skills: preliminary results of a survey of trainees completing internship training in Kuwait. Bulletin of the Kuwait Institute for Medical Specialization.

